# Association between long-term exposure to low ambient PM_2.5_ and cardiovascular hospital admissions: A UK Biobank study

**DOI:** 10.1016/j.envint.2024.109011

**Published:** 2024-10

**Authors:** Jacopo Vanoli, Jennifer K. Quint, Sanjay Rajagopalan, Massimo Stafoggia, Sadeer Al-Kindi, Malcolm N. Mistry, Pierre Masselot, Arturo de la Cruz Libardi, Chris Fook Sheng Ng, Lina Madaniyazi, Antonio Gasparrini

**Affiliations:** a School of Tropical Medicine and Global Health, Nagasaki University, Nagasaki, Japan; b Environment & Health Modelling (EHM) Lab, Department of Public Health Environments and Society, London School of Hygiene & Tropical Medicine, London, UK; c School of Public Health, Imperial College London, London, UK; d Harrington Heart and Vascular Institute, University Hospitals Cleveland Medical Center, Cleveland, OH 44106, United States; e Department of Epidemiology, Lazio Region Health Service ASL ROMA 1, Rome, Italy; f Center for Health and Nature, Houston Methodist, Houston, TX, United States; g Department of Economics, Ca’ Foscari University of Venice, Venice, Italy; h Department of Global Health Policy, Graduate School of Medicine, The University of Tokyo, Tokyo, Japan

**Keywords:** Air pollution, Cardiovascular disease, Hospital admissions, Long-term

## Abstract

**Introduction:**

A causal link between air pollution exposure and cardiovascular events has been suggested. However fewer studies have investigated the shape of the associations at low levels of air pollution and identified the most important temporal window of exposure. Here we assessed long-term associations between particulate matter < 2.5 µm (PM_2.5_) at low concentrations and multiple cardiovascular endpoints using the UK Biobank cohort.

**Methods:**

Using data on adults (aged > 40) from the UK Biobank cohort, we investigated the associations between 1-year, 3-year and 5-year time-varying averages of PM_2.5_ and incidence of major adverse cardiovascular events (MACE), myocardial infarction (MI), heart failure, atrial fibrillation and flutter and cardiac arrest. We also investigated outcome subtypes for MI and stroke. Events were defined as hospital inpatient admissions. We fitted Cox proportional hazard regression models applying extensive control for confounding at both individual and area level. Finally, we assessed the shape of the exposure–response functions to assess effects at low levels of exposure.

**Results:**

We analysed data from 377,736 study participants after exclusion of prevalent subjects. The average follow-up (2006–2021) was 12.9 years. We detected 19,353 cases of MACE, 6,562 of acute MI, 6,278 of heart failure, 1,258 for atrial fibrillation and flutter, and 16,327 for cardiac arrest. Using a 5-year exposure window, we detected positive associations (for 5 μg/m^3^ increase in PM_2.5_) for 5-point MACE of [1.12 (95 %CI: 1.00–1.26)], heart failure [1.22 (1.00–1.50)] and cardiac arrest [1.16 (1.03–1.31)]. We did not find any association with acute MI, while non-ST-elevation MI was associated with the 1-year exposure window [1.52 (1.12–2.07)]. The assessment of the shape of the exposure–response relationships suggested that risk is approximately linear for most of the outcomes.

**Conclusions:**

We found positive associations between long-term exposure to PM_2.5_ and multiple cardiovascular outcomes for different exposure windows. The cardiovascular risk tends to rise even at exposure concentrations below 12–15 μg/m^3^, indicating high risk below UK national and international thresholds.

## 1 Introduction

Historically, cardiovascular events have been among the most prominent contributors to the global burden of disease, causing 4.75 million deaths annually (Vaduganathan et al., 2022). Thanks to significant scientific progress in medical therapies, preventive measures, and increased public awareness over the years, high-income countries have experienced a decline in adverse clinical endpoints related to cardiovascular issues. However, globally, cardiovascular diseases continue to pose a substantial burden and remain a primary concern for national healthcare systems (Cheema et al., 2022).

Air pollution is a well-recognized risk factor for cardiovascular diseases and, among other air pollutants, PM_2.5_ (particulate matter with an aerodynamic diameter less than 2.5 μm) is known to be the most detrimental.

PM_2.5_ has been suggested to be causally related to cardiovascular disease and modulates its effects through a multitude of mechanisms, including progression of atherosclerosis and promotion of vulnerable plaque (Sanjay et al., 2018). Both acute plaque instability and chronic progression of plaque may ultimately result in the presentation of acute myocardial infarction and stroke. Therefore, considerable attention has been devoted to understanding the timelines of exposure to air pollution and resultant cardiovascular events (Al-Kindi et al., 2020, Crouse et al., 2020). There is also an emerging body of evidence linking antecedent exposure to air pollution with heart failure hospitalization and arrhythmias, including both atrial fibrillation and ventricular arrhythmias (de Bont et al., 2022). However, there is a paucity of studies on the relevant temporal windows of exposure (Crouse et al., 2020).

Further, studies that have evaluated a variety of composite outcomes are rare (Osborne et al., 2023), and the presence of non-linear effects, especially at lower levels of exposure, has been investigated in detail only in a few investigations with only one in Europe (Wolf et al., 2021, Danesh Yazdi et al., 2019, Bai et al., 2019).

In this study, we made use of the wealth of individual-level data present in UK Biobank (UKB) cohort, paired it with temporally resolved ambient PM_2.5_ exposure data, to assess risks of hospitalizations for several cardiovascular outcomes. We aimed to explore long-term associations at low levels of PM_2.5_ using time-varying averages at multiple temporal windows of exposure. We also assessed non-linear effects by varying the shape of the exposure–response function and by restricting the analysis to subjects with exposures below predefined thresholds.

## 2 Methods

### 2.1 Population (UK Biobank cohort)

The British prospective cohort study, UK Biobank (UKB), enrolled approximately half a million individuals aged 40 to 69 years between 2006 and 2010. As a first cohort assessment, the participants underwent an in-person visit in one of the 22 assessment centres located across Great Britain (England, Scotland, and Wales). The visit included multiple questionnaires regarding lifestyles and personal characteristics. Anthropometric measures and biological samples were also collected. Participants were followed up through periodical linkage with administrative health databases, including mortality and cancer national registries as well as primary and secondary care visits. The cohort profile has been described in detail in previous publications (Sudlow et al., 2015, Fry et al., 2017). Specific details regarding the UKB database can be found on the showcase website (https://biobank.ndph.ox.ac.uk/showcase/).

### 2.2 Study design

This study followed a time-to-event design. In this analysis, we excluded subjects with cardiovascular hospital admission prior to enrolment. Subsequently, we excluded subjects who, at enrolment time, self-reported prior cardiovascular diseases and/or hypertension medication. Participants were censored at the date of event occurrence, date of death, loss to follow-up, or the administrative end of follow-up (set here to 31/12/2021), whichever came first.

### 2.3 Exposure assessment and linkage

We assigned exposure at individual level combining PM_2.5_ predictions and residential history data. The original PM_2.5_ data were represented by daily levels predicted on a 1-km grid across the UK in the period 2003–2021 using a hybrid spatio-temporal machine learning (ML) model. The model used an ensemble of ML algorithms trained using ground monitor series and a series of spatial and spatio-temporal predictors, including outputs from emission-dispersion models, remote sensing satellite data, as well as land-use and traffic variables, among others (de la Cruz et al., 2024). The model performance, assessed using cross-validation, provided an overall coefficient of determination (R^2^) of 0.80 at daily scale. The residential data were available in the UKB database, including periods and geocoded locations with 100 m rounding. The data were validated internally, and the mobility history continuously updated through general practitioner registration or direct reporting by the participants.

The linkage process was performed in two steps. First, we constructed daily exposure series for each residential location by interpolating gridded exposure values using bilinear method. This approach consist of a linear interpolation over a two-dimensional grid and allowed preserving the exposure information while masking the original residential data, thus preventing back-tracing of the individual locations. Second, we composed the subject-specific exposure series by matching the daily series for corresponding residential periods. The process has been described in detail in a previous publication (Vanoli et al., 2024).

### 2.4 Hospital admissions outcomes

At the time of enrolment, people consented for access to a variety of personal information, including linked electronic health records and residential address locations. The UKB provides access to summary datasets including first inpatient hospital visits and operation codes. For each outcome, ICD-10 code and date of first primary or secondary diagnosis are made available. In this analysis, we used codes for the following outcome diagnoses: heart failure (I50.x, where “.x” defines all code subtypes), atrial fibrillation and flutter (I46, I46.0, I46.1, I46.9), cardiac arrest (I48, I48.0, I48.1, I48.2, I48.3,I 48.4), acute myocardial infarction (I21.x, I23.x), ST-elevation myocardial infarction (STEMI, I21.0–3), non-ST-elevation myocardial infarction (NSTEMI, I21.4), intracerebral stroke (I61.x), ischaemic stroke (I63.x, I64.x), and subarachnoid stroke (I60.x). In addition, we created a composite major adverse cardiovascular event (5-point MACE) outcome, defined as the occurrence of either acute MI (I21.x, I23.x), stroke (I60.x,I61.x,I63.x,I.64.x), unstable angina (I20.0) and heart failure (I50.x) and death due to cardiovascular disease (I00-I99). Details on the outcome diagnoses’ definitions can be found on the UKB website (https://biobank.ndph.ox.ac.uk/ukb/label.cgi?id = 2002).

### 2.5 Statistical analysis

We constructed separate cohorts to analyze each outcome based on an extended Cox proportional hazard model for time-varying exposures where the follow-up of each subject was split by calendar year. Therefore, we performed the analysis based on an extended Cox proportional hazard model with time-varying exposure (Andersen and Gill, 1982). We defined the model using calendar years as timescale and we stratified by assessment centre, sex, and year of birth, thus ensuring control for differential risks by age. The extended survival data was linked with annual exposure averages assigned over a lag window of five years, from lag 0 (the year of the event) until lag 4 (i.e. fourth year before the year of the event), consistently with previous studies (Crouse et al., 2015). Subjects with incomplete exposure history were excluded from the analysis.

In the main analysis, the exposure term was defined as the time-dependent average across the lag periods (in years) and we assumed a linear exposure–response relationship. Specifically, we investigated the associations for lag 0 (1-year average), lag 0–2 (3-year average) and lag 0–4 (5-year average) in separate models, due to their potentially high correlation. Additionally, we evaluated the shape of the association between time-varying PM_2.5_ for lag 0–4 and each outcome by estimating a non-linear response function using penalized splines, with the optimal degrees of freedom selected using the Akaike Information Criterion (AIC).

We a priori specified two confounder models: *Model 1* included the matching variables used for stratification (assessment centre, sex, and year of birth) and individual covariates determined at recruitment: ethnic background education level, household income, employment status, smoking status, packs of cigarettes per year, average alcohol intake per week, waist-to-hip ratio, physical activity (measured using the International Physical Activity Questionnaire (IPAQ) scale) and living alone (a proxy for marital status). In *Model 2*, we added control for area-level covariates, including the Townsend Deprivation Index measured in 2010, urban–rural classification (urban, town or fringe and village), and greenness percentage around the baseline residential address (at 1000 m buffer based on the UKB internal definition (Generalised Land Use Database Statistics for England, 2005, Morton et al., 2011)).

Estimates of the associations were reported as hazard ratios (HRs) for each hospitalization outcome per 5 μg/m^3^ increments in PM_2.5_, with 95 % confidence intervals (CI). Missing values in the baseline covariates were imputed using multiple imputation by chained equation (MICE), producing five imputed datasets, with estimates combined using Rubin’s rule (Barnard and Rubin, 1999).

### 2.6 Additional analyses

We performed a sensitivity analysis including only person-years assigned to exposure levels below 10 (WHO 2005 limits) or 12 µg/m^3^, as done previously (Wolf et al., 2021). We did not investigate associations at exposure levels below the WHO 2021 annual limits (5 µg/m^3^) because of the paucity of data in that exposure range. To evaluate sensitivity in the associations due to different MACE definitions, we conducted sensitivity analyses using 4- and 3-point MACE. Those were defined as the 5-point MACE outcome sequentially excluding diagnosis for heart failure (4-point MACE) and unstable angina (3-point MACE), respectively. To assess potential changes in the association attributable to the COVID-19 period we conducted an analysis with follow-up up to 31/12/2019. We conducted an additional analysis subsetting the cases only to the participants who had the diagnosis in the primary position. We also reported results by IQR increase (3.7 µg/m^3^) to make our results more in line with the exposure distribution. Finally, we performed an analysis including a washout period in order to account for potential healthy-volunteer and other selection biases, as recommended in a recent publication (Chen et al., 2024).

Data cleaning and statistical analyses were conducted using in R Statistical Software (version 4.2.1) and the following packages were used: data.table, survival, mice, parallel, ggplot2 and gridExtra.

## 3 Results

### 3.1 Descriptives

**Fig. 1:**
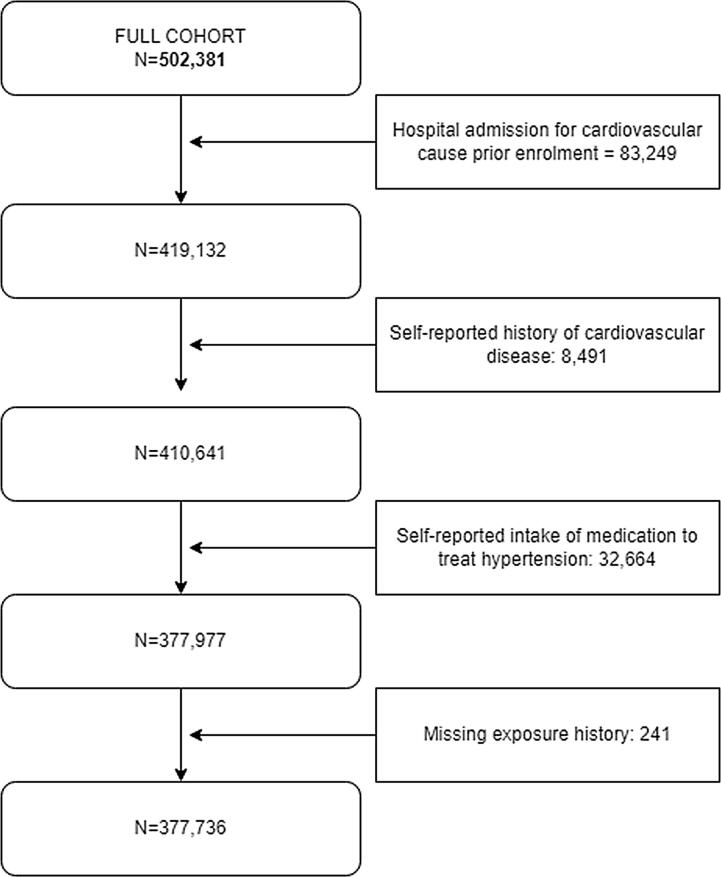
Flow diagram representing the selection of the sample of the UK Biobank.

The original dataset included 502,381 individuals. We excluded subjects with cardiovascular inpatient hospital admission prior to enrolment (n = 83,249), with self-reported history cardiovascular disease (n = 8,491) and hypertension medication (n = 32,664).Finally, 241 participants were excluded due to (partially) missing exposure histories, providing a final cohort of 377,736 individuals (Fig. 1). The participants were followed-up for an average of 12.9 years, with a total of 4,877,026 person-years. During the follow-up, among all the participants, 6,278 had inpatient hospital visit due to heart failure, 1,258 of atrial fibrillation and flutter, 16,327 of cardiac arrest, 6,562 of acute myocardial infarction, 2,710 of MI STEMI 2,426 of MI NSTEMI, 928 of intracerebral stroke, 4,526 of ischaemic stroke, 664 of subarachnoid stroke. For the composite outcomes, 5-point MACE status was reported for 19,353 participants.

The cohort had slightly more females than males (See Table 1), with an average age of 55 at baseline, and most of the cohort was of white ethnicity. More than 70 % of the subjects had at least received a diploma and 60 % were employed at the time of recruitment. About 11 % of participants were smokers, approximately half of the rate in the general UK population in 2011(General Lifestyle Survey:, 2011). Most subjects (84 %) lived in urban settings. In the proximity of the residential address, the average greenspace percentage and the average Townsend deprivation index were 45 and −1.39, respectively (See Table 1). These values reflect relatively wealthy residential surroundings.

**Table 1:** Descriptive statistics for continuous (mean with 5th-95th percentile range and missing) and categorical (counts/percentage) baseline characteristics and number of outcomes’ events in the UKB cohort.

sex	Female	211,843 (56.1 %)
	Male	165,893 (43.9 %)
	Missing (%)	0 (0.0 %)
ethnicity	White	356,597 (94.4 %)
	Other	19,767 (5.2 %)
	Missing (%)	1,372 (0.4 %)
employment status	Employed	235,367 (62.3 %)
	Retired	108,985 (28.9 %)
	Other	29,730 (7.9 %)
	Missing (%)	3,654 (1.0 %)
educational level	Low	54,078 (14.3 %)
	Professional Qualification	41,721 (11.0 %)
	Highschool diploma	144,801 (38.3 %)
	College/University degree	130,347 (34.5 %)
	Missing (%)	6,789 (1.8 %)
household income	Less than 18,000	64,043 (17.0 %)
	18,000 to 30,999	79,325 (21.0 %)
	31,000 to 51,999	88,037 (23.3 %)
	Greater than 100,000	19,863 (5.3 %)
	Missing (%)	54,165 (14.3 %)
physical activity (IPAQ score)	low	55,596 (14.7 %)
	moderate	125,054 (33.1 %)
	high	125,601 (33.3 %)
	Missing (%)	71,485 (18.9 %)
alcohol intake	Never	27,563 (7.3 %)
	Special occasions only	41,122 (10.9 %)
	One to three times a month	42,435 (11.2 %)
	Once or twice a week	98,996 (26.2 %)
	Three or four times a week	89,992 (23.8 %)
	Daily or almost daily	77,229 (20.4 %)
	Missing (%)	399 (0.1 %)
smoking status	Never	212,625 (56.3 %)
	Previous	123,450 (32.7 %)
	Current	40,332 (10.7 %)
	Missing (%)	1,329 (0.4 %)
living alone	No	308,249 (81.6 %)
	Yes	67,837 (18.0 %)
	Missing (%)	1,650 (0.4 %)
urban/rural classification	Urban	317,266 (84.0 %)
	Town/fringe	28,263 (7.5 %)
	Village/Rural	28,204 (7.5 %)
	Missing (%)	4,003 (1.1 %)
age at baseline	Years	55.46 (42.00 to 68.00)
	Missing (%)	0 (0.0 %)
waist-to-hip ratio		0.86 (0.72 to 1.01)
	Missing (%)	1,210 (0.3 %)
Smoking intensity	packs-year	7.27 (0.00 to 37.50)
	Missing (%)	57,592 (15.2 %)
Townsend deprivation index (2010		−1.39 (−5.06 to 4.77)
	Missing (%)	467 (0.1 %)
Greenspace	percentage	45.09 (15.40 to 87.38)
	Missing (%)	45,206 (12.0 %)

**Number of events**		
3-point MACE		14,087
4-point MACE		15,186
5-point MACE		19,353
acute MI		6,562
MI STEMI		2,710
MI NSTEMI		2,426
intracerebral stroke		928
ischaemic stroke		4,526
subarachnoid stroke		664
heart failure		6,278
atrial fibrillation and flutter		1,258
cardiac arrest		16,327

Figure S1 showed the box-and-whiskers plot of the distribution of annual averages of PM_2.5_ across the years from 2007 until 2021. The plot indicated that all UKB participants are permanently exposed to exposure values below the UK Air Quality Objectives (AQO) and EU Air Quality Directives (AQD) for 2015 and 2020 of 25 µg/m^3^. After a slight increase in 2011, the distribution of PM_2.5_ had generally declined over time. Since 2015, the majority of the cohort has been exposed to levels below the the 2005 WHO Air Quality Guidelines (AQG) limit of 10 µg/m^3^. Seldom annual exposure levels were below the new WHO AQG 2020 limits of 5 µg/m^3^. The correlation matrix (Table S7) between the exposure windows showed high to very high correlation among the exposure windows.

### 3.2 Associations between CVD and PM_2.5_ exposure

In Table 2, we showed the linear associations, reported as hazard ratios (HRs) for a 5 µg/m^3^ increase, between PM_2.5_ exposure with different lag windows and each cardiovascular outcome. In the fully-adjusted model (Model 2), the exposure was significantly associated with elevated risk for diagnosis of heart failure, intracerebral stroke, cardiac arrest and MI NSTEMI. For example, using an exposure window with lag 0–4, heart failure displayed an HR of 1.22 (95 %CI: 1.00–1.50) and intracerebral stroke of 1.94 (1.15–3.29). Associations were also positive for 5-point MACE (1.12 (1.00–1.26) at lag 0–4) but we found the strongest effects for shorter exposure windows (lag 0–2, 1.15 (1.03–1.28)). We did not find any evidence of linear associations with ischaemic stroke, subarachnoid stroke, acute MI, and atrial fibrillation and flutter. In general, associations for several outcomes were positive but did not reach statistical significance at the 5 % level, probably due to limited statistical power.

**Table 2:** Hazard ratios (HRs, with 95 % confidence intervals) of cardiovascular outcomes associated with an increase of 5 μg/m^3^ in PM_2.5_ in the UKB cohort, for combinations of length of exposure windows (lag0, lag02, lag04) and confounding control.

**Outcome**	**exposure window**	**Model 1**	**Model 2**
**5-point MACE**	lag0	1.17 (1.08–1.28)	1.13 (1.02–1.24)
	lag02	1.20 (1.09–1.32)	1.15 (1.03–1.28)
	lag04	1.18 (1.07–1.31)	1.12 (1.00–1.26)

**Myocardial Infarction (MI)**			
Acute	lag0	0.98 (0.84–1.14)	1.07 (0.90–1.27)
	lag02	0.95 (0.81–1.12)	1.06 (0.87–1.28)
	lag04	0.95 (0.81–1.13)	1.06 (0.87–1.29)
STEMI	lag0	0.89 (0.71–1.12)	0.98 (0.75–1.28)
	lag02	0.91 (0.71–1.17)	1.03 (0.77–1.38)
	lag04	0.94 (0.73–1.22)	1.09 (0.80–1.49)
NSTEMI	lag0	1.17 (0.90–1.51)	1.52 (1.12–2.07)
	lag02	1.04 (0.80–1.36)	1.35 (0.97–1.88)
	lag04	1.03 (0.78–1.35)	1.32 (0.95–1.84)

**Cerebrovascular disease and stroke**			
Intracerebral stroke	lag0	1.81 (1.20–2.72)	1.74 (1.09–2.78)
	lag02	1.93 (1.26–2.97)	1.91 (1.15–3.17)
	lag04	1.96 (1.26–3.05)	1.94 (1.15–3.29)
Ischaemic stroke	lag0	1.14 (0.95–1.37)	1.07 (0.87–1.32)
	lag02	1.16 (0.96–1.41)	1.08 (0.86–1.36)
	lag04	1.12 (0.91–1.36)	1.01 (0.80–1.28)
Subarachnoid stroke	lag0	1.02 (0.64–1.61)	0.94 (0.56–1.56)
	lag02	1.08 (0.66–1.76)	0.98 (0.56–1.74)
	lag04	1.12 (0.67–1.88)	1.03 (0.56–1.89)

**Other outcomes**			
Heart failure	lag0	1.35 (1.15–1.58)	1.19 (1.00–1.42)
	lag02	1.40 (1.18–1.65)	1.21 (1.00–1.48)
	lag04	1.41 (1.19–1.68)	1.22 (1.00–1.50)
Atrial fibrillation and flutter	lag0	1.43 (1.01–2.01)	1.39 (0.94–2.05)
	lag02	1.43 (0.99–2.07)	1.38 (0.90–2.12)
	lag04	1.34 (0.92–1.96)	1.26 (0.81–1.95)
Cardiac arrest	lag0	1.13 (1.03–1.24)	1.09 (0.98–1.22)
	lag02	1.17 (1.06–1.30)	1.15 (1.02–1.29)
	lag04	1.19 (1.07–1.31)	1.16 (1.03–1.31)

The comparison between Model 1 and 2 indicates that the inclusion of area-level confounders led to an attenuation of the estimates, except for MI NSTEMI, for which the associations increased.

Overall, we did not find important differences in the associations across different exposure windows, with some exceptions. For instance, the increased risk for MI NSTEMI was significant only when we considered the exposure of the last year (lag0). In contrast, for cardiac arrest, the lag0 window showed a weak relationship, while the other windows had stronger associations.

Linear associations in Table 3 and S3 compared the main associations with those estimated for subsets of person-years exposure to low concentrations (<=10 and <=12). The results mostly showed the strongest effects below a concentration of 10 µg/m^3^, but the associations below 12 are more difficult to interpret and show unclear patterns across the outcomes. This is likely due to the uneven distribution of PM_2.5_ (figure S1) across calendar years, which showed concentrations higher than 12 in the first years of exposure (up to 2015) when likely few events had occurred and therefore the corresponding estimates are more uncertain.

**Table 3:** Hazard ratios (HRs, with 95 % confidence intervals) of cardiovascular outcomes in exposure subset analysis in the UKB cohort. Exposure is defined as lag04 (5-years time-dependent average). Results are for an increase of 5 μg/m^3^ in PM_2.5_ using fully adjusted models (Model 2).

**Outcome**	**Exposure subset**		
	**<= 10**	**<= 12**	**Full analysis**
**5-point MACE**	1.05 (0.84–1.32)	1.16 (1.01–1.34)	1.12 (1.00–1.26)

**Myocardial Infarction (MI)**			
Acute	1.27 (0.90–1.81)	0.78 (0.53–1.17)	1.06 (0.87–1.29)
STEMI	1.20 (0.94–1.53)	1.07 (0.81–1.43)	1.09 (0.80–1.49)
NSTEMI	1.19 (0.68–2.07)	0.75 (0.25–2.22)	1.32 (0.95–1.84)

**Cerebrovascular disease and stroke**			
Intracerebral stroke	1.70 (1.01–2.86)	1.10 (0.89–1.36)	1.94 (1.15–3.29)
Ischaemic stroke	1.45 (1.00–2.10)	0.98 (0.85–1.14)	1.01 (0.80–1.28)
Subarachnoid stroke	0.98 (0.74–1.29)	1.33 (0.95–1.87)	1.03 (0.56–1.89)

**Other outcomes**			
Heart failure	1.51 (0.58–3.93)	1.13 (0.95–1.35)	1.22 (1.00–1.50)
Atrial fibrillation and flutter	0.58 (0.27–1.22)	1.22 (0.98–1.52)	1.26 (0.81–1.95)
Cardiac arrest	0.95 (0.55–1.64)	1.08 (0.93–1.25)	1.16 (1.03–1.31)

**Fig. 2:**
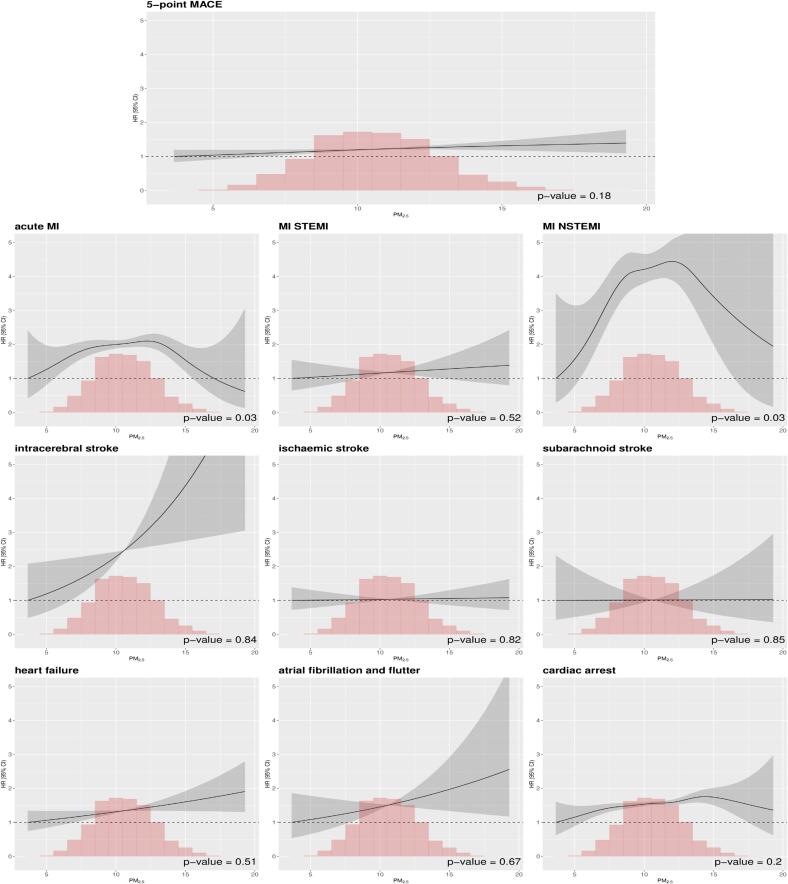
Concentration-response functions of the associations between lag04 (5-year time-dependent) of PM_2.5_ with cardiovascular events in the UK Biobank cohort. Models were fully adjusted (Model 2). The associations representing hazard ratios (HRs, with 95% confidence intervals) were estimated using penalized splines with degrees of freedom selected using the Akaike information criterion (AIC, solid line) with 95% confidence intervals (surrounding dashed lines).

Model allowing non-linear associations (Fig. 2) showed limited evidence for deviations from linear exposure–response function associations. For several outcomes, including 5-point MACE, MI STEMI, intracerebral stroke, heart failure, atrial fibrillation and flutter, and cardiac arrest, the penalized spline indicated linear associations, confirmed by the non-significant Wald test for non-linearity. In contrast, acute MI showed a non-linear effect, with the curve increasing up to 12–13 µg/m^3^ before declining. There was no evidence for a threshold in the association. No significant effects were observed for ischemic and subarachnoid stroke.

### 3.3 Additional analyses

Analysis of codes in only primary position (Table S1) showed similar associations with both primary and secondary code analysis (Table 2). Associations estimated with follow-up up to 31/12/2019 (Table S2) are mostly consistent with the main results (Table 2). In general, strong differences in the HR and confidence intervals between main and sensitivity analyses can be attributed to reduced sample sizes, considering that some outcomes only have a few hundred events in total and subsetting can lead to instabilities in the associations. Among MACE outcomes, 5-point MACE exhibited the highest and more precise associations compared to 3- and 4-point MACE (Table S4), likely due to increased statistical power.

For most of the outcomes, the exclusion of the wash-out period (Table S6) lead to stronger positive associations compared to the main linear analysis. Contrarily, subarachnoid and ischaemic stroke still displayed null effects.

## 4 Discussion

In this 15-year UK-based study, we used state-of-the-art epidemiological methods to assess the association between chronic exposure to time-varying PM_2.5_ at different yearly time windows and risk of hospitalizations for MACE and other severe clinical cardiovascular endpoints. We observed positive linear associations between PM_2.5_ across multiple exposure windows and several outcomes, including 5-point MACE, heart failure, intracerebral stroke and cardiac arrest. On the other hand, we found significant non-linear associations with overall acute MI and MI NSTEMI.

This study aimed at addressing research recommendations issued by the COMEAP 2019 report on air pollution and cardiovascular diseases (Kelly, 2019). First, the report highlighted the need for the use of more refined exposure estimates: in this analysis we applied highly resolved predictions from a state-of-the-art exposure model for the first time in the UK. Second, we investigated both major and subtypes of outcomes, non-linear effects and different exposure windows, with the purpose to shed more light on the mechanistic effects of long-term exposure on cardiovascular diseases, a question that was also part of the research recommendations.

### 4.1 MACE

This is one of the first studies to investigate the effect of long-term air pollution on MACE, and we found positive associations. Across all the time windows, UK resident adults living in areas with a 5 µg/m^3^ higher exposure experienced a 12 % to 15 % elevated risk of MACE-related hospitalizations compared to those in less exposed areas. The direction of the effect is in line with two studies on US veterans where a 9 % (by a 5 µg/m^3^ increase in PM_2.5_) increased risk was observed in individuals with prior coronary artery bypass grafting (Deo et al., 2024), and a 52 % increased risk in those with prior percutaneous coronary interventions (Motairek et al., 2023). In contrast, a Swedish (Carlsen et al., 2022) study showed no significant associations. It is important to consider that our studies differ in MACE composition, as our analysis included various clinical events, while, for example, the Swedish study focused on myocardial infarction and coronary interventions, making direct comparisons challenging. In our study, we attempted to include a simple and unambiguous MACE definition, therefore we decided to use the outcome as described in a recent literature review (Bosco et al., 2021). We hope that our choice may be of help to define outcomes in other medium-sized cohort studies that require composite definitions to conduct well-powered analyses.(Bosco et al., 2021).

#### 4.1.1 Myocardial infarction

Our findings on acute myocardial infarction (MI) show positive but not statistically significant associations [lag04: 1.07 (0.90–1.27)], aligning with two large *meta*-analyses (Alexeeff et al., 2021, Zhu et al., 2021) and a Canadian study (Bai et al., 2019). At low pollution levels, recent studies also reported positive effects (Wolf et al., 2021). However, while a Europe-based cohort reported similar associations with our study [1.02 (0.95–1.10)] (Wolf et al., 2021), US studies found stronger associations in the HR range of 1.13–1.188(Danesh Yazdi et al., 2019, Alexeeff et al., 2023).

This is one of the first long-term studies to examine associations with two types of myocardial infarction: STEMI and NSTEMI. Both outcomes were associated with the exposure, but we found no statistical significance with STEMI while there was a positive significant association with NSTEMI. The strong effects observed for the latter suggest that this subtype of MI may play a significant role in the overall association with acute MI in this cohort. A reason for this could be that NSTEMI is defined as partial blockages in a coronary artery, leading to less severe but more widespread myocardial ischemia. This condition makes it more vulnerable to factors that can worsen systemic inflammation and endothelial dysfunction, such as chronic exposure to air pollution. On the other hand, STEMI usually results from a complete and abrupt blockage of a coronary artery, causing a more acute event, which may be more associated with short-term air pollution (Newby et al., 2015, Kuźma et al., 2024). Even though the literature is not completely consistent in their distinction (STEMI and NSTEMI, 2024), our post-hoc hypothesis has been previously supported by the data as STEMI, but not NSTEMI, has been associated with short-term exposure (Gardner et al., 2014, Pope et al., 2015). There are currently no long-term studies considering their distinction. On the other hand, the use of a relatively young cohort (under 65–70 years) might also partially explain the increased risk of NSTEMI due to air pollution. While STEMI has traditionally been associated with younger individuals compared to NSTEMI, there has been a recent trend reversal in the UK, where since 2016, younger people (under 65) are more frequently admitted with NSTEMI than older individuals (MINAP and NAPCI, 2023). Additionally, NSTEMI cases are often underrepresented in hospitals due to their lower severity (How the NHS Cares for Patients with Heart Attack, 2015), suggesting that the actual numbers may be higher than reported for mid-aged adults. In this context, air pollution exposure may disproportionately increase the risk for younger individuals as they likely spend more time outdoor but have less severe outcomes. Therefore, while older adults are more vulnerable to air pollution effects, mid-age adults may be more at risk of slowly developing atherosclerotic plaques that lead to worse outcomes later in life, such as a case of STEMI. Supporting this, a recent large Polish study investigating mid-term (30 days) effects also found an increased risk of NSTEMI in younger individuals (under 65)(Kuźma et al., 2024).

Finally, a similarity between our investigation and the Polish study is that both analyses identified significant effects of mid-term air pollution exposure (ranging from one month to one year) on NSTEMI, indicating a potential impact of air pollution over this duration. However, they do not reveal differences substantial enough to support any significant post-hoc pathophysiological hypotheses. Nonetheless, these findings suggest that it may be valuable to incorporate sensitivity analyses with varying exposure windows in long-term studies, similar to those used in short-term research.

#### 4.1.2 Stroke

A large body of literature has covered the air pollution-stroke events relationship. In comparison to two *meta*-analyses that focused on general cerebrovascular disease (Alexeeff et al., 2021, Niu et al., 2021), our effects were higher for intracerebral stroke. Our analysis also indicates increased associations when compared with recent UK-(Cai et al., 2018);(Atkinson et al., 2013) and European-based (Wolf et al., 2021) studies. For example, Cai and colleagues (Cai et al., 2018), using the UKB cohort found null associations both for overall cerebrovascular diseases and stroke types (ischaemic and haemorrhagic). In this study we found heterogeneity of risk among stroke types. While several studies have assessed short-term associations, to our knowledge our study is among the first to investigate long-term effects on intracerebral stroke as a separate outcome (Verhoeven et al., 2021). This is relevant, as in the literature cerebrovascular events are often considered as a whole, although different stroke types are clinically considered as different diseases with separate etiologies (Verhoeven et al., 2021). There are physiological channels that connect exposure to air pollution might affect the occurrence of intracerebral stroke but no other types of haemorrhagic stroke: first, through known mechanisms, that is inflammation, oxidative stress, and endothelial dysfunction, fine particles effects may be more pronounced on the small vessels within the brain compared to other larger arteries. However, a previous MRI study did not find effects of PM on markers of small vessels disease (Power et al., 2018) and therefore the hypothesis is weak. Second, chronic particulate exposure may indirectly affect the brain through the autonomic respiratory reflex arcs as well as uptake of particles which can induce marked neuro-inflammation (Verhoeven et al., 2021). Finally, some authors hypothesized that overproduction of amyloid protein related to cerebral amyloid angiopathy may be the cause of intracerebral stroke (Wilker et al., 2018).

To conclude, the literature on the effects of air pollution on outcome subtypes is scattered and heterogeneous owing to the use of diverse study designs, exposure and outcome definitions and spatial differences in the particulate composition. More studies using state-of-the-art methodologies and harmonized datasets should be used to draw firmer hypotheses.

#### 4.1.3 Heart failure, atrial fibrillation and cardiac arrest

We detected significant between long-term air pollution and arrhythmias related-outcomes [for lag04: 1.13 (1.02–1.24)]. This is in line with recent investigations on atrial fibrillation on Medicare data (Mahdieh Danesh et al., 2021) and a Canadian cohort (Shin et al., 2019). The latter also assessed the shape of the exposure–response relationship, showing no evidence of an effect of PM_2.5_ below 6 µg/m^3^, in contrast with our findings. A *meta*-analytical estimate on four older studies showed a null association [0.96 (0.82–1.13) for 5 µg/m^3^ increase] (Pranata et al., 2020). Last, our associations for heart failure were positive, but on the boundaries of statistical significance[for lag04: 1.22 (1.00–1.50)], while a *meta*-analysis investigating the same outcome did not find effects. We found only one study (Shin et al., 2019) that examined hospital admissions related to atrial fibrillation and stroke, employing exposure windows similar to ours. No significant differences were observed for atrial fibrillation, whereas slight increases were noted for stroke for the 5-year window. However, our study suggests that the association may differ by exposure window.

#### 4.1.4 Windows of exposure

The minor difference in estimates suggests different windows influence only moderately the health risks. For instance, exposure closer to the event (lag0) has a stronger impact on the risk for MACE and MI NSTEMI. On the other hand, longer exposure windows (lag 0–2 and lag 0–4) can be more important for stroke and cardiac arrest. Our comparison of varying window widths was similar only to two previous studies (Crouse et al., 2020, Lefler et al., 2019) that mainly focused on the risk of premature mortality with mixed evidence. In Lefler and colleagues (Lefler et al., 2019), results did not highlight any relevant window of exposure for cardiopulmonary deaths, while in the study by Crouse and colleagues (Crouse et al., 2020), longer exposure windows were consistently associated with increased risk of mortality both for ischaemic heart and cerebrovascular disease.

#### 4.1.5 Shape of concentration–response function

The analysis of the concentration–response function suggest steep risks at concentrations even below 12–15 µg/m^3^, with no evidence of a threshold at the lowest values. This result highlights that despite the recent decreases in the air pollution levels, air pollution carries adverse effects even at very low levels and therefore new mitigation strategies are needed to account for the public health burden that cannot be avoided by further lowering concentrations. This finding contributes to a growing body of literature emphasizing the significance of addressing air pollution concerns not only at elevated levels but also at lower exposure levels (Wolf et al., 2021, Chen et al., 2023, Di et al., 2017). Furthermore, for MI our non-linear estimates detected increased risks below 12 µg/m^3^, agreeing with a previous study (Wolf et al., 2021). Contrarily, our results for stroke (intracerebral) suggest a linear relationship above 12 µg/m^3^, while previous investigations found stronger associations, especially at low levels (Wolf et al., 2021, Shin et al., 2019). This may be due to the choice of outcomes’ subtypes. Notably, the large majority of the previous literature focuses on US cohorts (Alexeeff et al., 2023, Di et al., 2017) while a few studies have investigated European cohorts (Wolf et al., 2021, Stafoggia et al., 2022).

### 4.2 Strengths and limitations

Our study carries several strengths. First, differently from the previous UK Biobank analyses of air pollution, this has been carried out using state-of-the-art exposure model with time-varying assignment, detailed confounders’ information and statistical methodologies analogously to the most relevant air pollution studies in the literature to date. One of our study’s main strengths lays in the utilization of time-dependent exposure summaries that enabled us to better define health risks compared to simpler exposure measures (Putter and van Houwelingen, 2017). This importantly distinguishes our approach from the majority of the UKB-based studies that solely incorporated fixed-time point exposures (Cai et al., 2018, Zhang et al., 2024, Luo et al., 2022, Parra et al., 2022) based on annual 2010 predictions of PM_2.5_. Another key strength is the use of the sizable UKB cohort with a rich history of individual data. This allowed us to include important individual-level confounders in the models that are usually unaccounted for in air pollution studies, such as smoking and waist-to-hip ratio.

Furthermore, in this study, we incorporated a composite outcome in addition to specific endpoints. One of the benefits of using a composite endpoint instead of individual ones is the increased statistical power, resulting from the inclusion of a larger number of cases. This is evident in some of the results for specific cardiovascular disease (CVD) endpoints, where hazard ratios (HRs) are elevated but did not achieve statistical significance. Additionally, using a broader CVD definition rather than specific endpoints may reduce outcome measurement errors.

We used specific outcome types (e.g., ischaemic stroke) instead of general definitions (e.g., cerebrovascular disease). The diversity of health effects revealed in this study, particularly when examining subtypes of outcomes, underscores the importance of defining more detailed outcomes, instead of using a wide range of ICD codes. Specificity may be crucial in assisting clinicians both to pinpoint events strongly linked to exposure to air pollution and investigate the pathophysiological mechanisms of the diseases.

Finally, the long observation period in contrast to the majority of studies on cardiovascular outcomes could also be the reason for the differences in estimates between our research and existing literature.

Some limitations in our study should be highlighted. The primary limitation of the UKB cohort is the potential lack of representativeness of the UK population, possibly including to healthy-volunteer bias (Fry et al., 2017). To mitigate this issue, in a sensitivity analysis we defined a wash-out period (Chen et al., 2024), excluding person-years up to 2013. The results showed higher health effects compared to the main analysis for certain outcomes, suggesting that the original estimates might be conservative. Second, the use of administrative ICD codes to assess outcomes can be misleading, leading to diagnosis misclassification due to lack of clinical details regarding the event (Verhoeven et al., 2021). However, previous research has validated codes for stroke and MI in the UK Biobank, showing 80–90 % positive predictive value (Woodfield et al., 2015). Third, the use of codes both in primary and secondary position could lead to associations biased upwards. This might occur if the hospital visit has a non-CV ICD code in primary position and a CV code of interest as secondary. If the code in primary position is positively associated with air pollution, consequently the resulting association with the CV code will also be inflated. However, our sensitivity analysis using only codes occurring in primary position showed only partial changes in the association for the majority of the outcomes. We did not use primary codes in the main analysis only due to a low number of cases. Moreover, for the same reason, particularly for outcomes sub-types such as stroke and MI, the corresponding main associations displayed large uncertainty, leading to several non-statistically significant results. Finally, we only investigated one pollutant without accounting for other important pollutants, such as NO_2_ and O_3_, known to be associated with health outcomes.

## 5 Conclusions

Our study suggests that long-term exposure to PM_2.5_ is associated with multiple cardiovascular outcomes. The strength of the associations did not significantly vary using different exposure windows. Consistently with the current literature, we found increased associations at low levels of exposure for the majority of the outcomes highlighting the importance of estimating exposure–response functions in long-term air pollution analyses. Finally, we found that selecting specific diagnoses instead of broad outcomes definitions may be beneficial to identify more relevant health outcomes.

## Funding

This work was supported by the Medical Research Council-UK (Grant ID: MR/Y003330/1), the European Union’s Horizon 2020 Project Exhaustion (Grant ID: 820655), Nagasaki University “Doctoral Program for World-leading Innovative and Smart Education” for Global Health (WISE), KENKYU SHIDO KEIHI (“the Research Grant”) and KYOIKU KENKYU SHIEN KEIHI (“the Stipend”).

## Declaration of Generative AI and AI-assisted technologies in the writing process

During the preparation of this work the author(s) used ChatGPT 3.5 to improve the language in some sentences in the Introduction and Discussion section. After using this tool/service, the author(s) reviewed and edited the content as needed and take(s) full responsibility for the content of the publication.

## CRediT authorship contribution statement

**Jacopo Vanoli:** Writing – original draft, Visualization, Software, Methodology, Investigation, Formal analysis, Data curation, Conceptualization. **Jennifer K. Quint:** Writing – review & editing, Supervision, Methodology, Conceptualization. **Sanjay Rajagopalan:** Writing – review & editing, Methodology, Conceptualization. **Massimo Stafoggia:** Writing – review & editing, Methodology, Conceptualization. **Sadeer Al-Kindi:** Writing – review & editing, Methodology, Conceptualization. **Malcolm N. Mistry:** Writing – review & editing. **Pierre Masselot:** Writing – review & editing. **Arturo de la Cruz Libardi:** Writing – review & editing. **Chris Fook Sheng Ng:** Writing – review & editing. **Lina Madaniyazi:** Writing – review & editing, Supervision, Conceptualization. **Antonio Gasparrini:** Writing – review & editing, Supervision, Resources, Project administration, Methodology, Funding acquisition, Data curation, Conceptualization.

## Declaration of competing interest

The authors declare that they have no known competing financial interests or personal relationships that could have appeared to influence the work reported in this paper.

## Data Availability

The authors do not have permission to share data.
