# Supplementary material for: Association between long-term exposure to low ambient PM2.5 and cardiovascular hospital admissions: A UK Biobank study
Source: Environ Int. 2024 Oct;192:109011. doi: 10.1016/j.envint.2024.109011 (PMC11496929; doi:10.1016/j.envint.2024.109011)

**~
Association between long-term exposure to low ambient PM_2.5_ and cardiovascular hospital admissions: a UK Biobank study**

Authors: Jacopo Vanoli^1,2^ , Jennifer K Quint^3^, Sanjay Rajagopalan^4^, Massimo Stafoggia^5^, Sadeer Al-Kindi^6^, Malcolm Mistry^2,7^, Pierre Masselot^2^, Arturo de la Cruz Libardi^2^, Chris Fook Sheng Ng^8^, Lina Madaniyazi^1^, Antonio Gasparrini^2^

^1^School of Tropical Medicine and Global Health, Nagasaki University, Nagasaki, Japan, ^2^ Environment & Health Modelling (EHM) Lab, Department of Public Health Environments and Society, London School of Hygiene & Tropical Medicine, London, UK, ^3^School of Public Health, Imperial College London, London, ^4^Harrington Heart and Vascular Institute, University Hospitals Cleveland Medical Center, Cleveland, OH, 44106, USA, ^5^Department of Epidemiology, Lazio Region Health Service ASL ROMA 1, Rome, Italy, ^6^Center for Health and Nature, Houston Methodist, Houston, Texas, United States, ^7^Department of Economics, Ca’ Foscari University of Venice, Venice, Italy, ^8^Department of Global Health Policy, Graduate School of Medicine, The University of Tokyo, Tokyo, Japan

Table S1: Hazard ratios (HRs, with 95% confidence intervals) of cardiovascular outcomes with ICD in primary position associated with an increase of 5 μg/m³ in PM_2.5_ in the UKB cohort, for combinations of length of exposure windows and confounding control.

| **Outcome** | **exposure window** | **Model 1** | **Model 2** |
| --- | --- | --- | --- |
|  |  |  |  |
| **5-point MACE** | lag0 | 1.13 (1.03-1.24) | 1.09 (0.98-1.22) |
|  | lag02 | 1.14 (1.03-1.26) | 1.10 (0.98-1.24) |
|  | lag04 | 1.13 (1.01-1.25) | 1.08 (0.95-1.22) |
|  |  |  |  |
| **Myocardial Infarction (MI)** | | | |
| Acute | lag0 | 0.93 (0.80-1.09) | 0.99 (0.83-1.18) |
|  | lag02 | 0.91 (0.77-1.07) | 0.96 (0.79-1.16) |
|  | lag04 | 0.91 (0.77-1.08) | 0.97 (0.79-1.18) |
| STEMI | lag0 | 0.83 (0.65-1.05) | 0.83 (0.64-1.09) |
|  | lag02 | 0.86 (0.67-1.11) | 0.88 (0.66-1.18) |
|  | lag04 | 0.90 (0.69-1.17) | 0.94 (0.69-1.27) |
| NSTEMI | lag0 | 1.12 (0.87-1.46) | 1.39 (1.02-1.89) |
|  | lag02 | 1.00 (0.76-1.32) | 1.22 (0.87-1.70) |
|  | lag04 | 0.98 (0.74-1.29) | 1.17 (0.84-1.64) |
|  |  |  |  |
| **Cerebrovascular disease and stroke** | | | |
| Intracerebral stroke | lag0 | 1.64 (1.05-2.54) | 1.63 (0.98-2.72) |
|  | lag02 | 1.81 (1.14-2.88) | 1.89 (1.09-3.29) |
|  | lag04 | 1.79 (1.11-2.89) | 1.87 (1.05-3.31) |
| Ischaemic stroke | lag0 | 1.22 (1.02-1.47) | 1.14 (0.92-1.40) |
|  | lag02 | 1.25 (1.03-1.52) | 1.15 (0.92-1.45) |
|  | lag04 | 1.20 (0.98-1.47) | 1.08 (0.86-1.37) |
| Subarachnoid stroke | lag0 | 1.14 (0.71-1.84) | 1.06 (0.62-1.81) |
|  | lag02 | 1.15 (0.69-1.92) | 1.05 (0.58-1.91) |
|  | lag04 | 1.12 (0.65-1.92) | 1.00 (0.53-1.88) |
|  |  |  |  |
| **Other outcomes** | | | |
| Heart failure | lag0 | 1.52 (1.14-2.03) | 1.26 (0.91-1.74) |
|  | lag02 | 1.59 (1.17-2.16) | 1.28 (0.90-1.83) |
|  | lag04 | 1.66 (1.21-2.28) | 1.34 (0.93-1.93) |
| Atrial fibrillation and flutter | lag0 | 2.85 (1.32-6.16) | 3.49 (1.46-8.38) |
|  | lag02 | 3.70 (1.65-8.28) | 5.17 (2.05-13.08) |
|  | lag04 | 3.88 (1.67-9.01) | 5.56 (2.10-14.72) |
| Cardiac arrest | lag0 | 1.12 (0.98-1.28) | 1.15 (0.98-1.34) |
|  | lag02 | 1.11 (0.96-1.28) | 1.14 (0.96-1.35) |
|  | lag04 | 1.12 (0.96-1.30) | 1.15 (0.96-1.37) |

Table S2: Hazard ratios (HRs, with 95% confidence intervals) of cardiovascular outcomes associated with an increase of 5 μg/m³ in PM_2.5_ in the UKB cohort with follow-up until 2019, for combinations of length of exposure windows and confounding control.

| **Outcome** | **exposure window** | **Model 1** | **Model 2** |
| --- | --- | --- | --- |
|  |  |  |  |
| **5-point MACE** | lag0 | 1.15 (1.06-1.26) | 1.11 (1.00-1.23) |
|  | lag02 | 1.17 (1.06-1.28) | 1.12 (1.00-1.24) |
|  | lag04 | 1.16 (1.05-1.28) | 1.10 (0.98-1.23) |
|  |  |  |  |
| **Myocardial Infarction (MI)** | | | |
| Acute | lag0 | 0.96 (0.83-1.11) | 1.06 (0.89-1.26) |
|  | lag02 | 0.94 (0.81-1.11) | 1.04 (0.86-1.25) |
|  | lag04 | 0.95 (0.80-1.12) | 1.05 (0.86-1.28) |
| STEMI | lag0 | 0.93 (0.74-1.17) | 1.04 (0.80-1.36) |
|  | lag02 | 0.93 (0.73-1.19) | 1.06 (0.80-1.42) |
|  | lag04 | 0.95 (0.74-1.23) | 1.11 (0.82-1.51) |
| NSTEMI | lag0 | 1.06 (0.83-1.36) | 1.39 (1.02-1.88) |
|  | lag02 | 1.00 (0.77-1.29) | 1.27 (0.92-1.75) |
|  | lag04 | 1.00 (0.77-1.31) | 1.28 (0.92-1.77) |
|  |  |  |  |
| **Cerebrovascular disease and stroke** | | | |
| Intracerebral stroke | lag0 | 1.72 (1.16-2.57) | 1.65 (1.03-2.64) |
|  | lag02 | 1.89 (1.25-2.88) | 1.88 (1.14-3.09) |
|  | lag04 | 1.90 (1.23-2.94) | 1.87 (1.11-3.15) |
| Ischaemic stroke | lag0 | 1.07 (0.90-1.29) | 0.99 (0.80-1.22) |
|  | lag02 | 1.11 (0.92-1.34) | 1.02 (0.82-1.27) |
|  | lag04 | 1.07 (0.88-1.31) | 0.97 (0.77-1.22) |
| Subarachnoid stroke | lag0 | 1.01 (0.64-1.58) | 0.91 (0.55-1.53) |
|  | lag02 | 1.07 (0.66-1.73) | 0.98 (0.56-1.71) |
|  | lag04 | 1.12 (0.67-1.87) | 1.03 (0.57-1.88) |
|  |  |  |  |
| **Other outcomes** | | | |
| Heart failure | lag0 | 1.37 (1.17-1.59) | 1.22 (1.01-1.46) |
|  | lag02 | 1.37 (1.17-1.61) | 1.20 (0.99-1.45) |
|  | lag04 | 1.41 (1.19-1.66) | 1.22 (1.00-1.49) |
| Atrial fibrillation and flutter | lag0 | 1.11 (1.00-1.23) | 1.10 (0.98-1.24) |
|  | lag02 | 1.13 (1.02-1.27) | 1.13 (0.99-1.28) |
|  | lag04 | 1.12 (1.00-1.25) | 1.10 (0.96-1.26) |
| Cardiac arrest | lag0 | 1.11 (1.00-1.23) | 1.09 (0.97-1.22) |
|  | lag02 | 1.13 (1.01-1.25) | 1.11 (0.98-1.25) |
|  | lag04 | 1.11 (0.99-1.24) | 1.08 (0.95-1.23) |

Table S3: Complete version of table 3 including all the length of the exposure window. Hazard ratios (HRs, with 95% confidence intervals) of cardiovascular outcomes in exposure subset analysis in the UKB cohort. Results are for an increase of 5 μg/m³ in PM_2.5_ using fully adjusted models (Model 2).

| **Outcome** | **exposure window** | **Exposure subset** | | |
| --- | --- | --- | --- | --- |
|  |  | **<= 10** | **<= 12** | **Full analysis** |
| **5-point MACE** | lag0 | 1.23 (1.03-1.46) | 1.16 (1.03-1.31) | 1.13 (1.02-1.24) |
|  | lag02 | 1.21 (0.98-1.48) | 1.20 (1.05-1.37) | 1.15 (1.03-1.28) |
|  | lag04 | 1.05 (0.84-1.32) | 1.16 (1.01-1.34) | 1.12 (1.00-1.26) |
|  |  |  |  |  |
| **Myocardial Infarction (MI)** | | | | |
| Acute | lag0 | 1.45 (1.11-1.90) | 1.04 (0.76-1.41) | 1.07 (0.90-1.27) |
|  | lag02 | 1.34 (0.97-1.84) | 1.01 (0.71-1.45) | 1.06 (0.87-1.28) |
|  | lag04 | 1.27 (0.90-1.81) | 0.78 (0.53-1.17) | 1.06 (0.87-1.29) |
| STEMI | lag0 | 1.26 (1.01-1.56) | 1.08 (0.84-1.38) | 0.98 (0.75-1.28) |
|  | lag02 | 1.19 (0.94-1.51) | 1.01 (0.77-1.34) | 1.03 (0.77-1.38) |
|  | lag04 | 1.20 (0.94-1.53) | 1.07 (0.81-1.43) | 1.09 (0.80-1.49) |
| NSTEMI | lag0 | 1.19 (0.79-1.79) | 0.88 (0.39-1.97) | 1.52 (1.12-2.07) |
|  | lag02 | 1.26 (0.77-2.08) | 0.89 (0.33-2.36) | 1.35 (0.97-1.88) |
|  | lag04 | 1.19 (0.68-2.07) | 0.75 (0.25-2.22) | 1.32 (0.95-1.84) |
|  |  |  |  |  |
| **Cerebrovascular disease and stroke** |  |  |  |  |
| Intracerebral stroke | lag0 | 1.94 (1.31-2.87) | 1.14 (0.97-1.33) | 1.74 (1.09-2.78) |
|  | lag02 | 1.68 (1.06-2.66) | 1.07 (0.88-1.30) | 1.91 (1.15-3.17) |
|  | lag04 | 1.70 (1.01-2.86) | 1.10 (0.89-1.36) | 1.94 (1.15-3.29) |
| Ischaemic stroke | lag0 | 1.63 (1.17-2.26) | 1.08 (0.95-1.22) | 1.07 (0.87-1.32) |
|  | lag02 | 1.48 (1.03-2.12) | 1.01 (0.88-1.16) | 1.08 (0.86-1.36) |
|  | lag04 | 1.45 (1.00-2.10) | 0.98 (0.85-1.14) | 1.01 (0.80-1.28) |
| Subarachnoid stroke | lag0 | 1.04 (0.85-1.29) | 1.28 (1.00-1.65) | 0.94 (0.56-1.56) |
|  | lag02 | 1.11 (0.86-1.42) | 1.32 (0.97-1.78) | 0.98 (0.56-1.74) |
|  | lag04 | 0.98 (0.74-1.29) | 1.33 (0.95-1.87) | 1.03 (0.56-1.89) |
|  |  |  |  |  |
| **Other outcomes** | | | | |
| Heart failure | lag0 | 1.62 (0.80-3.30) | 1.06 (0.93-1.21) | 1.19 (1.00-1.42) |
|  | lag02 | 2.06 (0.86-4.95) | 1.07 (0.92-1.25) | 1.21 (1.00-1.48) |
|  | lag04 | 1.51 (0.58-3.93) | 1.13 (0.95-1.35) | 1.22 (1.00-1.50) |
| Atrial fibrillation and flutter | lag0 | 0.95 (0.53-1.69) | 1.08 (0.92-1.27) | 1.39 (0.94-2.05) |
|  | lag02 | 0.85 (0.43-1.69) | 1.09 (0.90-1.33) | 1.38 (0.90-2.12) |
|  | lag04 | 0.58 (0.27-1.22) | 1.22 (0.98-1.52) | 1.26 (0.81-1.95) |
| Cardiac arrest | lag0 | 1.38 (0.86-2.21) | 1.06 (0.93-1.21) | 1.09 (0.98-1.22) |
|  | lag02 | 1.17 (0.69-1.98) | 1.12 (0.97-1.30) | 1.15 (1.02-1.29) |
|  | lag04 | 0.95 (0.55-1.64) | 1.08 (0.93-1.25) | 1.16 (1.03-1.31) |

Table S4: Hazard ratios (HRs, with 95% confidence intervals) of three MACE outcomes definitions associated with an increase of 5 μg/m³ in PM_2.5_ in the UKB cohort, for combinations of length of exposure windows and confounding control. *Results for 5-point MACE are the same as Table 2.

| **Outcome** | **exposure window** | **Model 1** | **Model 2** |
| --- | --- | --- | --- |
|  |  |  |  |
| **3-point MACE** | lag0 | 1.11 (1.00-1.23) | 1.10 (0.98-1.24) |
|  | lag02 | 1.13 (1.02-1.27) | 1.13 (0.99-1.28) |
|  | lag04 | 1.12 (1.00-1.25) | 1.10 (0.96-1.26) |
| **4-point MACE** | lag0 | 1.11 (1.00-1.23) | 1.09 (0.97-1.22) |
|  | lag02 | 1.13 (1.01-1.25) | 1.11 (0.98-1.25) |
|  | lag04 | 1.11 (0.99-1.24) | 1.08 (0.95-1.23) |
| **5-point MACE*** | lag0 | 1.17 (1.08-1.28) | 1.13 (1.02-1.24) |
|  | lag02 | 1.20 (1.09-1.32) | 1.15 (1.03-1.28) |
|  | lag04 | 1.18 (1.07-1.31) | - 1. 1.00-1.26) |

Table S5: Hazard ratios (HRs, with 95% confidence intervals) of cardiovascular outcomes associated with an increase of the IQR (3.7 μg/m³) in PM2.5 in the UKB cohort for combinations of length of exposure windows and confounding control.

| **Outcome** | **exposure window** | **Model 1** | **Model 2** |
| --- | --- | --- | --- |
|  |  |  |  |
| **5-point MACE** | lag0 | 1.13 (1.06-1.20) | 1.09 (1.01-1.18) |
|  | lag02 | 1.14 (1.07-1.23) | 1.11 (1.02-1.20) |
|  | lag04 | 1.13 (1.05-1.22) | 1.09 (1.00-1.18) |
|  |  |  |  |
| **Myocardial Infarction (MI)** | | | |
| Acute | lag0 | 0.98 (0.88-1.10) | 1.05 (0.93-1.20) |
|  | lag02 | 0.97 (0.86-1.09) | 1.04 (0.91-1.20) |
|  | lag04 | 0.97 (0.85-1.09) | 1.04 (0.90-1.21) |
| STEMI | lag0 | 0.92 (0.77-1.09) | 1.00 (0.82-1.21) |
|  | lag02 | 0.93 (0.78-1.12) | 1.04 (0.84-1.29) |
|  | lag04 | 0.95 (0.79-1.16) | 1.08 (0.86-1.36) |
| NSTEMI | lag0 | 1.12 (0.93-1.36) | 1.37 (1.09-1.72) |
|  | lag02 | 1.03 (0.85-1.26) | 1.26 (0.99-1.60) |
|  | lag04 | 1.02 (0.84-1.25) | 1.24 (0.97-1.58) |
|  |  |  |  |
| **Cerebrovascular disease and stroke** | | | |
| Intracerebral stroke | lag0 | 1.54 (1.14-2.08) | 1.49 (1.05-2.11) |
|  | lag02 | 1.62 (1.18-2.22) | 1.60 (1.09-2.33) |
|  | lag04 | 1.63 (1.18-2.27) | 1.62 (1.10-2.39) |
| Ischaemic stroke | lag0 | 1.10 (0.96-1.26) | 1.06 (0.90-1.23) |
|  | lag02 | 1.12 (0.97-1.29) | 1.07 (0.90-1.26) |
|  | lag04 | 1.09 (0.94-1.26) | 1.02 (0.86-1.21) |
| Subarachnoid stroke | lag0 | 1.01 (0.72-1.42) | 0.94 (0.65-1.38) |
|  | lag02 | 1.05 (0.73-1.52) | 0.98 (0.64-1.48) |
|  | lag04 | 1.09 (0.74-1.59) | 1.01 (0.64-1.58) |
|  |  |  |  |
| **Other outcomes** | | | |
| Heart failure | lag0 | 1.25 (1.11-1.40) | 1.14 (1.00-1.30) |
|  | lag02 | 1.28 (1.13-1.45) | 1.16 (1.00-1.34) |
|  | lag04 | 1.29 (1.14-1.47) | 1.16 (1.00-1.35) |
| Atrial fibrillation and flutter | lag0 | 1.30 (1.01-1.68) | 1.33 (0.99-1.78) |
|  | lag02 | 1.31 (1.00-1.71) | 1.34 (0.97-1.84) |
|  | lag04 | 1.24 (0.94-1.64) | 1.25 (0.90-1.73) |
| Cardiac arrest | lag0 | 1.10 (1.02-1.17) | 1.07 (0.99-1.16) |
|  | lag02 | 1.13 (1.05-1.22) | 1.11 (1.02-1.21) |
|  | lag04 | 1.14 (1.05-1.23) | 1.11 (1.02-1.22) |

Table S6: Hazard ratios (HRs, with 95% confidence intervals) of cardiovascular outcomes associated with an increase of 5 μg/m³ in PM2.5 in the UKB cohort excluding the *wash-out* period from enrolment until 2013 for combinations of length of exposure windows and confounding control.

| **Outcome** | **exposure window** | **Model 1** | **Model 2** |
| --- | --- | --- | --- |
|  |  |  |  |
| **5-point MACE** | lag0 | 1.24 (1.12-1.38) | 1.20 (1.06-1.36) |
|  | lag02 | 1.26 (1.13-1.41) | 1.22 (1.07-1.38) |
|  | lag04 | 1.25 (1.12-1.39) | 1.20 (1.05-1.36) |
|  |  |  |  |
| **Myocardial Infarction (MI)** | | | |
| Acute | lag0 | 1.15 (0.95-1.39) | 1.31 (1.05-1.63) |
|  | lag02 | 1.12 (0.92-1.36) | 1.29 (1.02-1.63) |
|  | lag04 | 1.12 (0.93-1.37) | 1.30 (1.03-1.64) |
| STEMI | lag0 | 1.13 (0.85-1.51) | 1.28 (0.91-1.79) |
|  | lag02 | 1.19 (0.88-1.61) | 1.40 (0.97-2.00) |
|  | lag04 | 1.22 (0.90-1.66) | 1.45 (1.01-2.09) |
| NSTEMI | lag0 | 1.14 (0.87-1.49) | 1.46 (1.06-2.02) |
|  | lag02 | 1.03 (0.78-1.36) | 1.31 (0.93-1.84) |
|  | lag04 | 1.03 (0.78-1.35) | 1.29 (0.92-1.80) |
|  |  |  |  |
| **Cerebrovascular disease and stroke** | | | |
| Intracerebral stroke | lag0 | 1.68 (1.05-2.68) | 1.77 (1.03-3.06) |
|  | lag02 | 1.76 (1.08-2.86) | 1.92 (1.07-3.43) |
|  | lag04 | 1.75 (1.07-2.84) | 1.91 (1.06-3.42) |
| Ischaemic stroke | lag0 | 1.12 (0.90-1.40) | 1.07 (0.83-1.37) |
|  | lag02 | 1.11 (0.89-1.39) | 1.04 (0.80-1.36) |
|  | lag04 | 1.09 (0.87-1.36) | 1.01 (0.77-1.31) |
| Subarachnoid stroke | lag0 | 0.89 (0.50-1.57) | 0.84 (0.44-1.60) |
|  | lag02 | 0.97 (0.53-1.77) | 0.94 (0.47-1.89) |
|  | lag04 | 1.02 (0.56-1.88) | 1.00 (0.49-2.05) |
|  |  |  |  |
| **Other outcomes** | | | |
| Heart failure | lag0 | 1.41 (1.18-1.69) | 1.23 (1.00-1.51) |
|  | lag02 | 1.51 (1.25-1.81) | 1.31 (1.05-1.64) |
|  | lag04 | 1.50 (1.25-1.81) | 1.30 (1.05-1.63) |
| Atrial fibrillation and flutter | lag0 | 1.51 (1.01-2.24) | 1.55 (0.98-2.46) |
|  | lag02 | 1.50 (0.99-2.27) | 1.54 (0.94-2.51) |
|  | lag04 | 1.40 (0.92-2.12) | 1.40 (0.86-2.28) |
| Cardiac arrest | lag0 | 1.17 (1.05-1.31) | 1.15 (1.01-1.30) |
|  | lag02 | 1.22 (1.08-1.36) | 1.20 (1.05-1.38) |
|  | lag04 | 1.23 (1.09-1.38) | 1.21 (1.06-1.39) |

Table S7: Correlations between different exposure windows.

| exposure windows | lag0 | lag02 | lag04 |
| --- | --- | --- | --- |
| lag0 | 1 |  |  |
| lag02 | 0.93 | 1 |  |
| lag04 | 0.84 | 0.96 | 1 |

Figure S1: Distribution of annual average exposure to PM_2.5_ across participants of the UKB cohort in the period 2003-2021, with limits corresponding to air quality guidelines/directives (AQG and AQD) from the World Health Organization (WHO), European Union (EU), and the United Kingdom (UK).


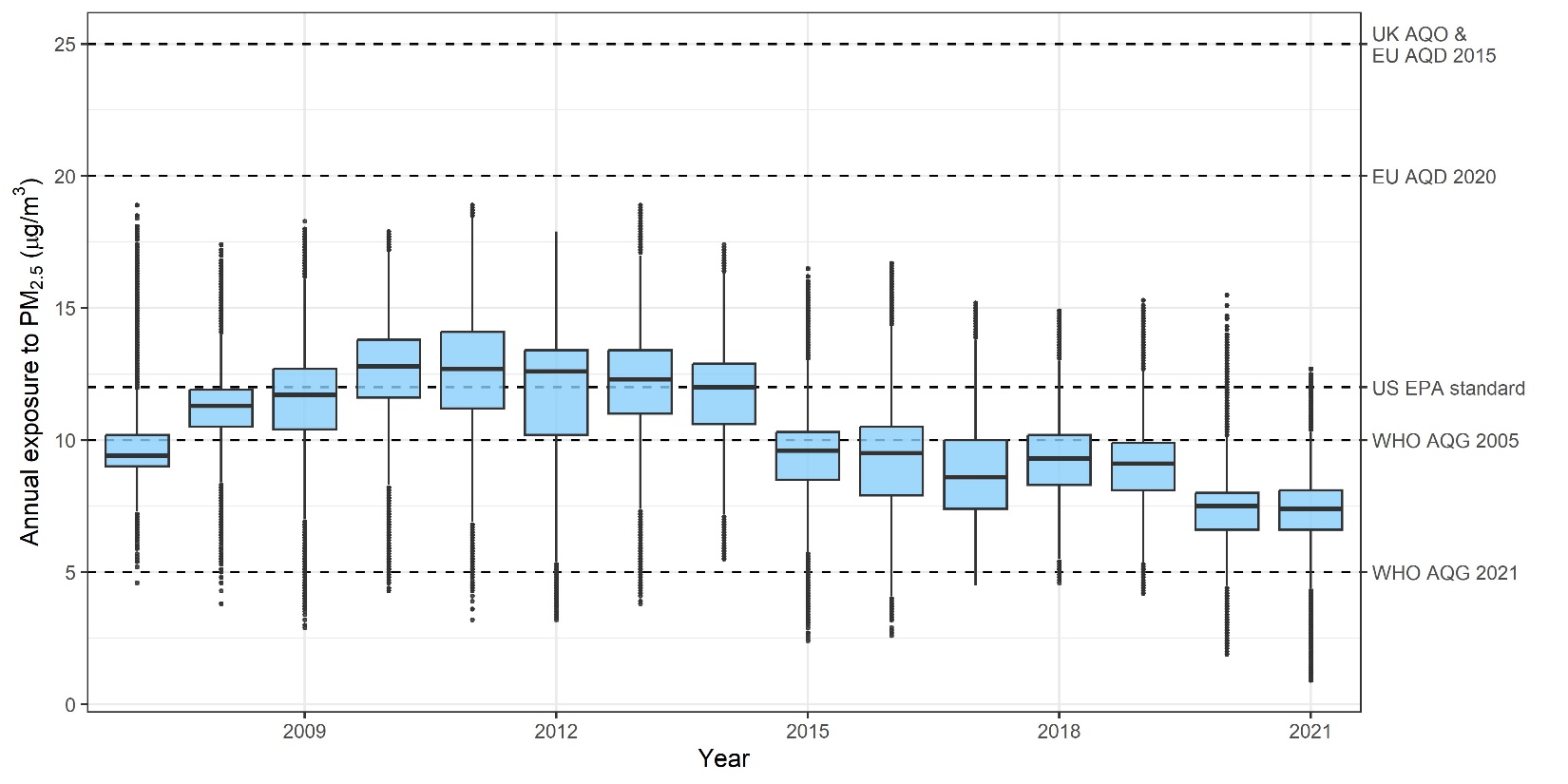

Supplement: Supplementary Data 1 [file mmc1.docx]
